# Management of complex renal cysts in Canada: results of a survey study

**DOI:** 10.1186/s12894-020-00614-5

**Published:** 2020-04-28

**Authors:** Félix Couture, Antonio Finelli, Amélie Tétu, Bimal Bhindi, Rodney H. Breau, Anil Kapoor, Wassim Kassouf, Luke Lavallée, Simon Tanguay, Philippe D. Violette, Patrick O. Richard

**Affiliations:** 1 Division of Urology, Department of Surgery, Centre Hospitalier Universitaire de Sherbrooke, 3001, 12e avenue N, Sherbrooke, Quebec, J1H 5N4 Canada; 2 Division of Urology, Department of Surgery, Princess Margaret Cancer Centre, University Health Network and the University of Toronto, Toronto, Canada; 3 Unité de recherche clinique et épidémiologique, Centre de Recherche, Centre Hospitalier Universitaire de Sherbrooke, Sherbrooke, Canada; 4 Division of Urology, Department of Surgery, Southern Alberta Institute of Urology, Calgary, Canada; 5 Division of Urology, Department of Surgery, Ottawa Hospital, Ottawa Hospital Research Institute and University of Ottawa, Ottawa, Canada; 6 Division of Urology, Department of Surgery, Juravinski Hospital, St. Joseph Healthcare, McMaster University, Hamilton, Canada; 7 Division of Urology, Department of Surgery, McGill University Health Centre, McGill University, Montreal, Canada; 8 Division of Urology, Department of Surgery, Woodstock Hospital, Woodstock, Canada

**Keywords:** Complex renal cysts, Bosniak, Active surveillance, Surgery, Management

## Abstract

**Background:**

Bosniak III and IV cysts have a high risk of malignancy and have traditionally been managed surgically. However, growing evidence suggests that many can be managed by active surveillance. The main objective of this study was to characterize the use of surveillance in the management of complex renal cysts.

**Methods:**

A web-based survey was sent to all registered, active members of the Canadian Urological Association (*N* = 583) in October 2018.

**Results:**

The survey response rate was 24.7%. Management of Bosniak III cysts varied considerably. A large proportion of respondents (33.1%) offered active surveillance in > 50% of cases. Only 13.7% of respondents reported never or rarely (< 5% of cases) offering surveillance. In contrast, for Bosniak IV cysts, 60.1% of urologists never or rarely offered surveillance, while only 10.1% offer it in > 50% of cases. A significantly greater proportion of academic urologists, compared to non-academic urologists, viewed surveillance as a management option for patients with a Bosniak III or IV cyst. The most commonly reported barriers to a greater adoption of surveillance were concerns regarding its oncologic safety, the lack of data to support surveillance in this population, and the lack of triggers for discontinuation of active surveillance and intervention.

**Conclusions:**

Despite active surveillance being included as a management option in guidelines, many Canadian urologists are reluctant to offer surveillance to patients with Bosniak III or IV cysts. Practice patterns are heterogeneous among those offering surveillance. High-quality studies are required to better define the benefits and risks of cystic renal mass surveillance.

## Background

Up to one third of individuals over 60 years of age will be diagnosed with a renal cyst following abdominal imaging [1]. Renal cysts are classified according to the Bosniak classification, which categorizes the cysts according to their degree of complexity and risk of malignancy [2–4]. Cystic renal cell carcinoma represents approximately 5–10% of all renal malignancies [5]. Bosniak III and IV cysts have a high risk of malignancy (40–60% and 80–90%, respectively) and have traditionally been managed with surgical excision [6, 7]. However, similar to small non-cystic renal masses, there is growing evidence suggesting that most of these cysts are indolent and unlikely to metastasize [5, 8–15]. Thus, active surveillance has been proposed as an alternative to surgery [6, 16].

Recent observational data has provided support for the use of active surveillance among patients with complex renal cysts [17, 18]. However, the adoption of this treatment strategy for the management of complex cysts in Canada and the criteria used by urologists as triggers for discontinuation of surveillance and intervention have yet to be defined.

The objectives of this survey study were to characterize the use of active surveillance in the management of complex renal cysts in Canada and to elicit the perceived barriers to adoption. We also aimed to characterize patient and disease factors associated with use of active surveillance and triggers used as criteria for intervention.

## Methods

This is a descriptive cross-sectional study using electronic surveys and conducted based on known guidelines [19, 20]. Following approval from the CIUSSS de l’Estrie - CHUS Research Ethics Board, a pilot questionnaire was developed and tested among 20 urologists in October 2018. All items were then revised according to the feedback received in the pilot survey. The survey questions were formatted as short answer, multiple choice, or Likert rating scale questions. Responses were anonymous and no personal information was collected or stored. An electronic open survey was generated on REDcap™ and distributed via email. The survey was distributed to all active members of the Canadian Urological Association (CUA) with a functional email address. Three emails (one initial and two reminders) containing a link to the English language questionnaire were sent out to all 583 members between October 30 and November 19, 2018. Questions covered practice patterns regarding complex renal cysts, criteria used to select candidates for active surveillance, triggers to intervene in patients on active surveillance, and perceived barriers to a greater adoption of active surveillance (Additional file 1). In analyses, we excluded non-practicing urologists, urologists who reported not managing complex renal cysts, and urologists who gave incomplete demographic information or who did not answer questions beyond the demographic section.

Continuous and categorical variables were reported using medians (interquartile range [IQR]) and proportions, respectively. Chi-squared tests were used to assess differences between specific groups of respondents (types of practice). Statistical analyses were conducted using SAS/STAT® software, version 9.4. All statistical tests were two-sided and *p*-values < 0.05 were considered statistically significant.

## Results

In total, 144 urologists (24.7%) responded to the survey. From these, we excluded three urologists who were not actively in practice or did not manage complex cysts, and two urologists because they did not answer any questions other than demographics. Therefore, our study included 139 respondents, of which 88.8% (*N* = 122) answered every survey question.

Demographic data are presented in Table 1. Of the eligible respondents, 71 (51.1%) practiced in an academic center, while 68 (48.9%) practiced in a non-academic setting. The majority (87.0%) of respondents reported managing ≤20 new complex renal cysts on an annual basis.

**Table 1:** Demographic data of the included respondents (*N* = 139)

Variables	N (%)
**Years in independent practice**	
●1 to 5	56 (40.3)
●6 to 10	29 (20.9)
●11 to 15	21 (15.1)
● > 15	33 (23.7)
**Fellowship training**	
●Urologic oncology	46 (33.1)
●Endourology/Minimally invasive surgery	30 (21.6)
●Other fellowship	23 (16.6)
●No fellowship training	40 (28.8)
**Type of practice**	
●Academic hospital	71 (51.1)
●Community or rural hospital	66 (47.5)
●Office-based practice	2 (1.4)
**Area of practice**	
●British Columbia	10 (7.2)
●Prairies	18 (13.0)
●Ontario	59 (42.5)
●Quebec	43 (30.9)
●Atlantic Canada	9 (6.5)
**Annual number of new complex renal cysts cases (Bosniak III-IV)**	
●1–5	24 (17.3)
●6–10	42 (30.2)
●11–20	55 (39.6)
●21–30	10 (7.2)
● > 30	8 (5.8)

### Use of active surveillance

**Fig. 1:**
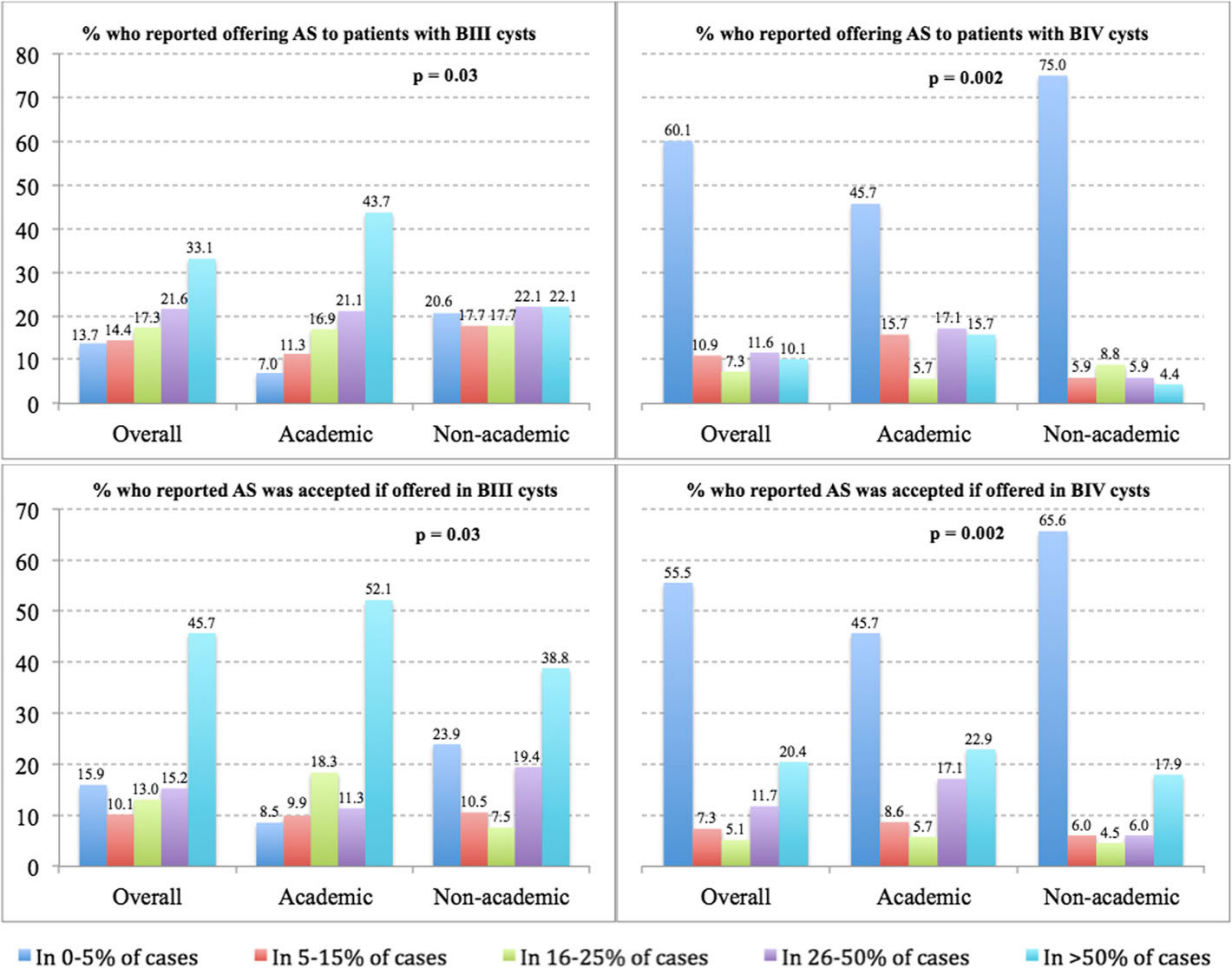
Active surveillance use – Percentage of respondents who reported offering active surveillance (AS) to a certain proportion of patients and percentage of these patients they felt accept surveillance, for Bosniak III (BIII) and for Bosniak (BIV) cysts (overall and according to type of practice)

Of the eligible respondents, 13.7% of urologists never or rarely (< 5% of cases) offered active surveillance, while 33.1% offered active surveillance in > 50% of patients with a Bosniak III cysts in whom surgical excision is considered a suitable treatment option (Fig. 1). When compared to non-academic urologists, a significantly greater proportion of academic urologists offered active surveillance as a treatment option to their patients with a Bosniak III cyst. When patients with a Bosniak III cyst were offered active surveillance as a treatment option, it was perceived by nearly half of the urologists (45.7%) that this option was accepted in a majority of cases. The likelihood of a patient accepting active surveillance for the management of a Bosniak III cyst was perceived to be significantly greater by urologists from academic centres than by urologists from non-academic centres.

Unlike for Bosniak III cyst, a majority of urologists (60.1%) never or rarely (< 5% of cases) offered active surveillance for Bosniak IV cysts, while only 10.1% offered active surveillance in > 50% of cases. A significantly greater proportion of academic than non-academic urologists viewed active surveillance as a viable treatment alternative for patients with a Bosniak IV cyst. However, even when active surveillance was proposed to a patient as a treatment option, only 20.4% of urologists stated that this option was accepted by > 50% of patients.

### Barriers to active surveillance adoption

Several potential barriers to a greater adoption of active surveillance were noted (Table 2). The most commonly reported ones were [1] the patient’s and physician’s concerns regarding the oncologic safety and/or benefits of active surveillance (89.8%), [2] the lack of data supporting active surveillance in patients with Bosniak III-IV cysts (74.2%), and [3] the lack of specific triggers for intervention during surveillance for complex renal cysts (75.8%).

**Table 2:** Perceived barriers to a more widespread adoption of active surveillance for Bosniak III-IV cysts (*N* = 128)

Concerns	Disagree N (%)	Neither agree nor disagree N (%)	Agree N (%)
1) Patient and physician concerns regarding the oncologic safety and/or benefits of active surveillance.	8 (6.3)	5 (3.9)	114 (89.8)
2) The psychological burden for the physicians or patients	33 (25.8)	29 (22.7)	66 (51.6)
3) The belief that active surveillance is not an appropriate alternative since an effective surgical option already exists.	43 (33.6)	31 (24.2)	54 (42.2)
4) The lack of data to support active surveillance in patients with BIII-IV	17 (13.3)	16 (12.5)	95 (74.2)
5) The lack of specific triggers for intervention during active surveillance for cystic tumors	11 (8.6)	20 (15.6)	97 (75.8)
6) The lack of guidance/knowledge/decision-aid tool on how to best manage and follow patients on active surveillance	22 (17.2)	30 (23.4)	76 (59.4)
7) The belief that active surveillance is not an efficient trade-off to surgery because it increases the burden of care (i.e., more visits and repeated tests).	69 (53.9)	36 (28.1)	23 (18.0)
8) The reliability of patients and the possibility of patients being lost to follow-up on active surveillance.	47 (36.7)	48 (37.5)	33 (25.8)

### Factors increasing likelihood of offering active surveillance

For both Bosniak III and IV cysts, age and the presence of comorbidities were perceived as having an impact on the likelihood of a patient being offered active surveillance as a treatment alternative (Table 3). The majority of urologists viewed older patients (> 75 years of age) as being ideal candidates. Nevertheless, nearly 30% of urologists thought that the lower age cut-off should be 65 years old for patients with Bosniak III cysts. Likewise, most urologists viewed that cyst size influenced their decision to offer surveillance and that cysts < 4 cm were ideal for active surveillance.

**Table 3:** Association of patient and tumor factors with likelihood of recommending active surveillance and specific cut-offs viewed as most appropriate for active surveillance (*N* = 127 for Bosniak III; *N* = 124 for Bosniak IV)

	Significant impact on likelihood to recommend active surveillance reported N (%)	
Characteristics	Bosniak III	Bosniak IV
**Patient factors**		
▪Age	121 (95.3)	104 (84.5)
*●Cutoff*		
*○ > 55 years old*	6 (5.0)	3 (2.9)
*○ > 65 years old*	35 (28.9)	17 (16.4)
*○ > 75 years old*	80 (66.1)	84 (80.8)
▪Presence of comorbidities	121 (96.0)	105 (85.4)
**Tumor factors**		
▪Cyst size	72 (56.7)	59 (48.0)
*●Cutoff*		
*○ < 4 cm*	56 (77.8)	54 (91.5)
*○ < 7 cm*	16 (22.2)	5 (8.5)
▪Size of nodular component	N/A	86 (69.9)
*●Upper limit cutoff, median (IQR)*		2 cm (1–3 cm)
▪Number of septa/calcification	32 (25.2)	25 (20.7)
*●Cutoff*		
*○ ≤ 2*	7 (22.9)	9 (36.0)
*○ ≤ 3*	20 (62.5)	12 (48.0)
*○ ≤ 4 or 5*	5 (15.7)	4 (16.0)
▪Thickness of septa/calcification	56 (44.1)	31 (25.2)
*●Upper limit cutoff, median (IQR)*	3 mm (2–5 mm)	5 mm (3–5 mm)
▪Cyst wall nodularity	95 (74.8)	N/A

More specifically, for Bosniak III cysts, the presence of cyst wall nodularity (74.8%) and the maximal thickness of septa/calcification (44.1%) were also considered characteristics that impacted the likelihood of offering active surveillance. An upper limit threshold of 3 mm (IQR 2–5 mm) in maximal thickness of septa/calcification was perceived as being most appropriate for surveillance. For Bosniak IV cysts, the size of the nodular component was seen as important to the decision to offer active surveillance (69.9%) with a median perceived upper limit cut-off of 2 cm (IQR 1–3 cm).

### Triggers for intervention during active surveillance

Several characteristics were perceived by urologists as being criteria for intervention for patients initially managed by active surveillance (Table 4). The most commonly reported criteria for Bosniak III cysts were [1] progression on imaging from Bosniak III to IV cysts, [2] worsening or change in the wall or septa enhancement, [3] progression or development of cyst wall nodularity. For Bosniak IV cysts, the two most common triggers for intervention were perceived as being [1] the growth rate of solid component (> 0.5 cm/year) and [2] the growth of solid component (> 3 cm).

**Table 4:** Criteria perceived as being triggers for intervention

	Criterion reported as being a trigger for intervention N (%)	
Characteristics	Bosniak III	Bosniak IV
Progression on imaging from Bosniak III to IV	110 (79.1)	N/A
Growth rate of solid component above threshold (for example: > 0.5 cm/year)	N/A	85 (61.2)
Growth of solid component above threshold (for example: > 3 cm)	N/A	88 (63.3)
Growth rate of cysts above threshold (for example: > 0.5 cm/year)	48 (34.5)	28 (20.1)
Doubling time of calculated volume ≤ 12 months	39 (28.1)	39 (28.1)
Progression in the number of septa or calcifications	29 (20.9)	28 (20.1)
Progression in the thickness of septa or calcifications	60 (43.2)	43 (30.9)
Worsening or change in the wall or septa enhancement	70 (50.4)	N/A
Progression or development of cyst wall nodularity	94 (67.6)	N/A
None of the above	2 (1.4)	3 (2.2)
I do not offer active surveillance	1 (0.7)	25 (18.0)

### Surgical management

When managed surgically, over half of respondents (57.3%) were more likely to offer a minimally invasive partial nephrectomy approach to patients with a complex cyst as the surgical management of choice. No significant differences in terms of surgical management were observed between academic and non-academic urologists (Additional file 2). When compared to the management of small non-cystic renal masses, the majority of surveyed urologists managed Bosniak III and IV cysts in a similar fashion to how they manage small non-cystic renal masses. However, 20.7% of respondents were more inclined to offer an open surgical approach, while 14.6% were more inclined to perform a radical nephrectomy as opposed to a partial nephrectomy. Again, there were no statistically significant differences observed in the management of academic and community urologists (Additional file 3).

## Discussion

Indirect evidence from the small non-cystic renal mass literature has supported the role of active surveillance as a management option for complex renal cysts [5, 8, 10, 11, 16, 21–24]. Two recent retrospective studies have also reported the outcomes of patients with a complex renal cyst who opted to be managed by active surveillance [17, 18]. The average cyst sizes in the two studies were 4.1 cm and 3.5 cm for Bosniak III cysts, and 3.1 cm and 3.8 cm for Bosniak IV lesions, respectively. Both studies have suggested that this approach could be safely used in this population, with only one death due to kidney cancer observed after 5 years of follow-up in these studies. Moreover, only two patients with a Bosniak IV cyst developed a metastasis out of 243 patients with a Bosniak III or IV cyst (0.8%) – both of whom had refused surgery despite evidence of local progression. Criteria for lesion progression included increase in cyst size, increase in vascularity, and increase in size of the solid component. Importantly, during the 5-year observation period, 65% of patients avoided surgery given the absence of lesion progression, and among patients who progressed, 16.5% were found to have a benign tumor on final pathology. While these results are encouraging, given the low quality of existing evidence, current guidelines on the management of complex renal cysts continue to recommend surgery as the mainstay treatment and suggest that the use of active surveillance should be reserved for select patients [6, 16].

This study sought out to assess the adoption of active surveillance in Canada and to examine barriers to more widespread use. We found that approximately one third of Canadian urologists stated that they offered active surveillance as a treatment option in greater than 50% of patients who are diagnosed with a Bosniak III cyst, while only 10% of urologists offered surveillance in the majority of Bosniak IV cases. Importantly, over 60% of urologists did not consider or rarely considered active surveillance as a treatment option for a Bosniak IV cyst. Furthermore, the adoption of active surveillance for both Bosniak III and IV cysts was significantly greater among academic urologists compared to non-academic urologists. Likely reflecting the confidence of urologists for active surveillance, a greater proportion of urologists felt that, when offered to a patient, the chance of surveillance being chosen as management strategy was greater among patients diagnosed with a Bosniak III cyst than those with a Bosniak IV cyst.

When respondents were questioned on potential barriers preventing a more widespread use of active surveillance, the most common perceived concerns were [1] the lack of data supporting this strategy in this population, [2] the oncologic safety and benefits of active surveillance and [3] the lack of guidance on how to perform active surveillance and which specific triggers should be used to recommend discontinuation of active surveillance. We asked respondents what patient and tumor characteristics increased their likelihood of recommending active surveillance. As expected, a number of factors seemed to influence the urologist’s decision, highlighting the fact that treatment decision for Bosniak III and IV cysts is a challenging one. We identified that a personal or familial history of kidney cancer, as well as the patient’s treatment preference, influenced the likelihood that a physician would offer active surveillance. Multiple tumor characteristics also seemed to influence the likelihood of offering surveillance, with the most reported features being cyst size, size of nodular component, and presence of cyst wall nodularity. Importantly, the respondents also identified criteria perceived as triggers to offer discontinuation of active surveillance. For Bosniak III, the most common triggers were progression of cyst on imaging from Bosniak III to IV or development of cyst wall nodularity, and worsening or change in the wall or septa enhancement. For Bosniak IV cysts, the most common criteria identified were growth rate of solid component and overall growth of solid component. Interestingly, for both Bosniak III and IV cysts, the growth of the cystic component was not considered by most urologists as being worrisome enough to warrant treatment.

Although this study offers insight into the current management of complex renal cysts in Canada, it is not devoid of limitations. First, the results are based on Canadian urologists’ perceptions, and the identified criteria for initiation and discontinuation of active surveillance have never been properly studied. Therefore, the reported use of active surveillance may not necessarily be generalized to all clinicians’ real-life practice. The reported patterns of active surveillance may provide a starting point for future studies, but criteria need to be validated before being applied in clinical practice. Second, there might be a selection bias in our cohort, as urologists interested in active surveillance of complex cysts may have responded to the survey more readily.

On the other hand, the study carries several strengths. First, the survey was pilot tested and validated by 20 experts in the field of urology. Second, the response rate to this survey was 24%, which is similar, and even higher than in other Canadian urology surveys [25, 26]. Third, this study was designed, conducted and reported according to appropriate recommendations for survey research [19, 20]. Most of the questions were structured in a closed format (binary, ordinal, nominal) in order to lower the bias of the responses.

## Conclusions

This study supports that, despite the lack of high-quality evidence, many Canadian urologists offer active surveillance as an option to patients with complex renal cysts, especially if they have a Bosniak III cyst. However, the lack of sufficient data or guidelines on safety seems to prevent widespread adoption of active surveillance. Prospective studies should be conducted to provide evidence on the oncologic safety, benefits, harms, cost, eligibility criteria, and guidelines for the discontinuation of active surveillance in the management of Bosniak III and IV cysts.

## Supplementary information

**Additional file 1.** Survey questions.

**Additional file 2.** Surgical management of choice for a patient with a Bosniak III or IV cyst (*N* = 131).

**Additional file 3.** Surgical management of complex cysts compared to the surgical management of solid small renal masses (*N* = 130).

## Data Availability

The datasets used and/or analysed during the current study are available from the corresponding author on reasonable request.

## Abbreviation

IQR: Interquartile range

## References

[1] Marumo K, Horiguchi Y, Nakagawa K, Oya M, Ohigashi T, Asakura H (2003). Incidence and growth pattern of simple cysts of the kidney in patients with asymptomatic microscopic hematuria. Int J Urol.

[2] Bosniak M (1986). The current radiological approach to renal cysts. Radiology..

[3] Bosniak M (1997). Diagnosis and Management of Patients with complicated cystic lesions of the kidney. AJR..

[4] Israel GM, Bosniak MA (2005). An update on the Bosniak renal cyst classification system. Urology..

[5] Bhatt JR, Jewett M, Richard PO, Kawaguchi S, Timilshina N, Evans A (2016). Multilocular cystic renal cell carcinoma: pathological T staging makes no difference to favorable outcomes and should be reclassified. J Urol.

[6] Richard PO, Violette PD, Jewett MAS, Pouliot F, Leveridge MJ, So A (2017). CUA guideline on the management of cystic renal lesions. Can Urol Assoc J.

[7] Schoots IG, Zaccai K, Hunink MG, Verhagen P (2017). Bosniak classification for complex renal cysts reevaluated: a systematic review. J Urol.

[8] Donin NM, Mohan S, Pham H, Chandarana H, Doshi A, Deng F-M (2015). Clinicopathologic outcomes of cystic renal cell carcinoma. Clin Genitourin Cancer.

[9] Gong K, Zhang N, He Z, Zhou L, Lin G, Na Y (2008). Multilocular cystic renal cell carcinoma: an experience of clinical management for 31 cases. J Cancer Res Clin Oncol.

[10] Nassir A, Jollimore J, Gupta R, Bell D, Norman R (2002). Multilocular cystic renal cell carcinoma: a series of 12 cases and review of the literature. Urology..

[11] Winters BR, Gore JL, Holt SK, Harper JD, Lin DW, Wright JL (2015). Cystic renal cell carcinoma carries an excellent prognosis regardless of tumor size. Urol Oncol.

[12] Bielsa O, Lloreta J, Gelabert-Mas A (1998). Cystic renal cell carcinoma: pathological features, survival and implications for treatment. Br J Urol.

[13] Corica FA, Iczkowski KA, Cheng L, Zincke H, Blute ML, Wendel A (1999). Cystic renal cell carcinoma is cured by resection: a study of 24 cases with long-term followup. J Urol.

[14] Koga S, Nishikido M, Hayashi T, Matsuya F, Saito Y, Kanetake H (2000). Outcome of surgery in cystic renal cell carcinoma. Urology..

[15] Murad T, Komaiko W, Oyasu R, Bauer K (1991). Multilocular cystic renal cell carcinoma. Am J Clin Pathol.

[16] Ljungberg B, Albiges L, Abu-Ghanem Y, Bensalah K, Dabestani S, Fernandez-Pello S, et al. European Association of Urology Guidelines on Renal Cell Carcinoma: The 2019 Update. Eur Urol. 2019;75(5):799–810. 10.1016/j.eururo.2019.02.011 30803729

[17] Chandrasekar T, Ahmad A, Fadaak K, Jhaveri K, Bhatt J, Jewett M (2018). Natural history of complex renal cysts: clinical evidence supporting active surveillance. J Urol.

[18] Pruthi D, Liu Q, Kirkpatrick I, Gelfond J, Drachenberg D (2018). Long-term surveillance of complex cystic renal masses and heterogeneity of Bosniak 3 lesions. J Urol.

[19] Burns KE, Duffett M, Kho ME, Meade MO, Adhikari NK, Sinuff T (2008). A guide for the design and conduct of self-administered surveys of clinicians. CMAJ..

[20] Eysenbach G (2004). Improving the quality of web surveys: the checklist for reporting results of internet E-surveys (CHERRIES). J Med Internet Res.

[21] Bhindi B, Thompson R, Lohse C, Mason R, Frank I, Costello B (2018). The probability of aggressive versus indolent histology based on renal tumor size: implications for surveillance and treatment. Eur Urol.

[22] Finelli A, Ismaila N, Bro B, Durack J, Eggener S, Evans A (2017). Management of small renal masses: American Society of Clinical Oncology clinical practice guideline. J Clin Oncol.

[23] Jewett MA, Mattar K, Basiuk J, Morash CG, Pautler SE, Siemens DR (2011). Active surveillance of small renal masses: progression patterns of early stage kidney cancer. Eur Urol.

[24] Pierorazio PM, Johnson MH, Ball MW, Gorin MA, Trock BJ, Chang P (2015). Five-year analysis of a multi-institutional prospective clinical trial of delayed intervention and surveillance for small renal masses: the DISSRM registry. Eur Urol.

[25] Richard PO, Martin L, Lavallee LT, Violette PD, Komisarenko M, Evans AJ (2018). Identifying the use and barriers to the adoption of renal tumour biopsy in the management of small renal masses. Can Urol Assoc J.

[26] Millar AC, Elterman DS, Goldenberg L, Van Asseldonk B, Curtis A, Jarvi K (2016). A survey of Canadian urologists' opinions and prescribing patterns of testosterone replacement therapy in men on active surveillance for low-risk prostate cancer. Can Urol Assoc J.

