# Supplementary material for: Management of complex renal cysts in Canada: results of a survey study
Source: BMC Urol. 2020 Apr 28;20:47. doi: 10.1186/s12894-020-00614-5 (PMC7189683; doi:10.1186/s12894-020-00614-5)
Supplement: Supplementary file 1 — Additional file 1. Survey questions. [file 12894_2020_614_MOESM1_ESM.docx]

**Appendix 1: Survey questions**

**A. Demographic & Practice**

A.1. – How many years have you been in independent practice as a urologist? _____

A.2. - What best describes your post-residency training? I have a fellowship in:

1. Urologic oncology
2. Endourology/Minimally invasive surgery
3. No fellowship training
4. Other

A.3. - What best describes your type of practice? I work in a:

1. Academic Hospital
2. Community Hospital
3. Office-based practice
4. Other

A.4. – What best describes the region where you are currently practicing?

1. West Coast (British Columbia)
2. Prairies (Alberta, Saskatchewan and Manitoba)
3. Ontario
4. Quebec
5. Atlantic Canada (NB, NS, PEI, Nfld)
6. Northern Canada (Yukon, Northwest Territories, Nunavut)

A.5. - On an annual basis, approximately how many new patients are referred to you for the management of a newly diagnosed complex renal cyst (Bosniak III-IV)?

a) None

b) 1-5

c) 6-10

d) 11-20

e) 21-30

f) > 30

**B. Bosniak III and IV cyst management**

B.1. – Of the patients referred to you with Bosniak cysts in which surgical extirpation is an option, to which proportion of patients do you offer active surveillance?

| **For Bosniak III:**  a) I do not offer AS  b) < 5%  c) 5-15%  d) 16-25%  e) 26-50%  f) > 50% | **For Bosniak IV:**  a) I do not offer AS  b) < 5%  c) 5-15%  d) 16-25%  e) 26-50%  f) > 50% |
| --- | --- |

B.2. – Of the patients who are referred to you with **Bosniak cysts and who are offered active surveillance,** what proportion of patients is **managed** by active surveillance?

| **For Bosniak III:**  a) I do not offer AS  b) < 5%  c) 5-15%  d) 16-25%  e) 26-50%  f) > 50% | **For Bosniak IV:**  a) I do not offer AS  b) < 5%  c) 5-15%  d) 16-25%  e) 26-50%  f) > 50% |
| --- | --- |

B.3. - On a scale of 0-100, what would be an acceptable cancer-specific mortality trade-off between active surveillance and surgery of a complex cyst? For example, I would be willing to accept **up to XX% additional risk** of death at 5 years by choosing active surveillance over definitive treatment.

B.4. – In patients with a Bosniak III or IV cyst undergoing surgery, are you more inclined to offer:

1. Open partial nephrectomy
2. Open radical nephrectomy
3. Minimally invasive partial nephrectomy
4. Minimally invasive radical nephrectomy

B.5. In general, is your surgical management for Bosniak III or IV cysts different from the one for small renal masses?

1. Yes, I tend to do more **radical nephrectomies** for complex cysts than I do for small renal masses
2. Yes, I tend to do more **open surgeries** for complex cysts than I do for small renal masses
3. Yes, I tend to do more **radical nephrectomies** and **open surgeries** for complex cysts than I do for small renal masses
4. No

**C. Barriers to adoption of AS**

C.1. - Which of the following do you believe are barriers to more widespread adoption of active surveillance in Bosniak III-IV cysts?

| **Barriers** | **Strongly disagree** | **Disagree** | **Neither agree nor disagree** | **Agree** | **Strongly agree** |
| --- | --- | --- | --- | --- | --- |
| 1. Patient’s or physician’s concerns regarding the oncologic safety and/or benefits of active surveillance for patients with complex cysts. |  |  |  |  |  |
| 2) The psychological burden for the physicians or patients. |  |  |  |  |  |
| 3) I don’t see the need to suggest active surveillance because we can offer an effective surgical treatment. |  |  |  |  |  |
| 4) The lack of data to support active surveillance in patients with Bosniak III-IV cysts. |  |  |  |  |  |
| 5) The lack of specific triggers for intervention during active surveillance for cystic tumours. |  |  |  |  |  |
| 6) The lack of guidance/knowledge/decision-aid tool on how to best manage and follow patients on active surveillance. |  |  |  |  |  |
| 7) Active surveillance is not an efficient trade-off to surgery because it increases the burden of care (i.e., more visits and repeated tests). |  |  |  |  |  |
| 8) I’m worried about the possibility of loss to follow-up during surveillance |  |  |  |  |  |

**D. Management of active surveillance**

D.1. – Do you consider the following factors in your decision to recommend active surveillance to a patient with a Bosniak III cyst?

D.1.1. Age: Yes No

If yes, which of the following criteria **increase** your willingness to offer active surveillance to a patient with a Bosniak III cyst?

- - 1. > 55
    2. > 65
    3. > 75

D.1.2. Cyst size: Yes No

If yes, which upper limit cut-off (cm) would you consider as appropriate to active surveillance in patients with a **Bosniak III** cyst? __________ cm

D.1.3. Presence of comorbidities: Yes No

D.1.4. Number of septa or calcifications: Yes No

If yes, which upper limit cut-off would you consider as appropriate to active surveillance in patients with a **Bosniak III** cyst?

- - 1. ≤ 1
    2. ≤ 2
    3. ≤ 3
    4. ≤ 4
    5. ≤ 5

D.1.5. Thickness of septa or calcification: Yes No

If yes, which upper limit cut-off (cm) would you consider as appropriate to active surveillance in patients with a **Bosniak III** cyst? __________ cm

D.1.6. Cyst wall nodularity: Yes No

D.2. - Do you consider the following factors in your decision to recommend active surveillance to a patient with a Bosniak IV cyst?

D.2.1. Age: Yes No

If yes, which of the following criteria **increase** your willingness to offer active surveillance to a patient with a Bosniak III cyst?

- - 1. > 55
    2. > 65
    3. > 75

D.2.2.Cyst size: Yes No

If yes, which upper limit cut-off (cm) would you consider as appropriate to active surveillance in patients with a **Bosniak III** cyst? __________ cm

D.2.3. Size of solid nodular component: Yes No

If yes, which upper limit cut-off (cm) would you consider as appropriate to active surveillance in patients with a **Bosniak III** cyst? __________ cm

D.2.4. Presence of comorbidities: Yes No

D.2.5. Number of septa or calcifications: Yes No

If yes, which upper limit cut-off would you consider as appropriate to active surveillance in patients with a **Bosniak III** cyst?

- - 1. ≤ 1
    2. ≤ 2
    3. ≤ 3
    4. ≤ 4
    5. ≤ 5

D.2.6. Thickness of septa or calcifications: Yes No

If yes, which upper limit cut-off (cm) would you consider as appropriate to active surveillance in patients with a **Bosniak III** cyst? __________ cm

D.3. - For patients with a Bosniak III cyst on active surveillance, what do you believe should be criteria for an intervention (besides patients’ choice and occurrence of metastasis)?

1. Progression on imaging from Bosniak III to IV
2. Growth rate of cysts above threshold (for example: > 0.5 cm/year)
3. Doubling time of calculated volume ≤ 12 months
4. Progression in the number of septa or calcifications
5. Progression in the thickness of septa or calcifications
6. Worsening or change in the wall or septa enhancement
7. None of the above
8. Not applicable: I do not offer active surveillance to patients with a Bosniak III cyst
9. Other: ____________________________________

D.4. - For patients with a Bosniak IV cyst on active surveillance, what do you believe should be criteria for an intervention (besides patients’ choice and occurrence of metastasis)?

1. Growth rate of solid component above threshold (for example: > 0.5 cm/year)
2. Growth of solid component above threshold (for example: > 3 cm)
3. Growth rate of cysts above threshold (for example: > 0.5 cm/year)
4. Doubling time of calculated volume ≤ 12 months
5. Progression in the number of septa or calcifications
6. Progression in the thickness of septa or calcifications
7. None of the above
8. Not applicable: I do not offer active surveillance to patients with a Bosniak IV cyst
9. Other: ___________________________________
