# Supplementary material for: Management of complex renal cysts in Canada: results of a survey study
Source: BMC Urol. 2020 Apr 28;20:47. doi: 10.1186/s12894-020-00614-5 (PMC7189683; doi:10.1186/s12894-020-00614-5)
Supplement: Supplementary file 2 — Additional file 2. Surgical management of choice for a patient with a Bosniak III or IV cyst (N = 131). [file 12894_2020_614_MOESM2_ESM.docx]

**Appendix 2: Surgical management of choice for a patient with a Bosniak III or IV cyst (N=131)**

| **Surgery** | **Overall, N (%)** | **Academic, N (%)** | **Community, N (%)** | **P value** |
| --- | --- | --- | --- | --- |
| **MIS partial** | 75 (57.3) | 42 (60.0) | 33 (54.1) | 0.3 |
| **MIS radical** | 13 (9.9) | 4 (5.7) | 9 (14.8) |  |
| **Open partial** | 42 (32.1) | 23 (32.9) | 19 (31.2) |  |
| **Open radical** | 1 (0.8) | 1 (1.4) | 0 |  |
