# Supplementary material for: Management of complex renal cysts in Canada: results of a survey study
Source: BMC Urol. 2020 Apr 28;20:47. doi: 10.1186/s12894-020-00614-5 (PMC7189683; doi:10.1186/s12894-020-00614-5)
Supplement: Supplementary file 3 — Additional file 3. Surgical management of complex cysts compared to the surgical management of solid small renal masses (N = 130). [file 12894_2020_614_MOESM3_ESM.docx]

**Appendix 3. Surgical management of complex cysts compared to the surgical management of solid small renal masses (N=130)**

| **Management** | **Overall, N (%)** | **Academic, N (%)** | **Community, N (%)** | **P value** |
| --- | --- | --- | --- | --- |
| **Same** | 92 (70.1) | 47 (68.2) | 45 (73.8) | 0.9 |
| **More Open** | 27 (20.7) | 15 (21.7) | 12 (19.7) |  |
| **More Radical** | 19 (14.6) | 10 (14.5) | 9 (14.8) |  |
